# Low perceived social support predicts later depression but not social phobia in middle adolescence

**DOI:** 10.1080/21642850.2014.966716

**Published:** 2014-10-28

**Authors:** Juha-Matti Väänänen, Mauri Marttunen, Mika Helminen, Riittakerttu Kaltiala-Heino

**Affiliations:** ^a^ Department of Adolescent Psychiatry, Tampere University Hospital, Tampere, Finland; ^b^ Department of Psychiatry, University of Helsinki, Helsinki, Finland; ^c^ Department of Adolescent Psychiatry, Helsinki University Central Hospital, Helsinki, Finland; ^d^ Department of Mental Health and Substance Use Services, National Institute for Health and Welfare, Helsinki, Finland; ^e^ Science Center, Pirkanmaa Hospital District, Tampere, Finland; ^f^ Tampere School of Public Health, University of Tampere, Tampere, Finland; ^g^ Medical School, University of Tampere, Tampere, Finland

**Keywords:** social anxiety, phobia, depression, social support, adolescents, gender differences, family support, peer support

## Abstract

Social phobia and depression are common and highly comorbid disorders in adolescence. There is a lack of studies on possible psychosocial shared risk factors for these disorders. The current study examined if low social support is a shared risk factor for both disorders among adolescent girls and boys. This study is a part of the Adolescent Mental Health Cohort Study's two-year follow-up. We studied cross-sectional and longitudinal associations of perceived social support with social phobia, depression, and comorbid social phobia and depression among girls and boys. The study sample consisted of 2070 15-year-old adolescents at baseline. Depression was measured by the 13-item Beck Depression Inventory, social phobia by the Social Phobia Inventory (SPIN), and perceived social support by the Perceived Social Support Scale-Revised (PSSS-R). Girls reported higher scores on the PSSS-R than boys in total scores and in friend and significant other subscales. Cross-sectional PSSS-R scores were lower among adolescents with social phobia, depression, and comorbid disorder than among those without these disorders. Low PSSS-R total score and significant other subscale were risk factors for depression among both genders, and low support from friends among girls only. Low perceived social support from any source was not a risk factor for social phobia or comorbid social phobia and depression. As conclusion of the study, low perceived social support was a risk factor for depression, but not a shared risk factor for depression and social phobia. Interventions enhancing perceived social support should be an important issue in treatment of depression.

## Introduction

Social phobia and depression are common disorders among adolescents. The point prevalence estimates of social phobia in adolescence range from 1.6% to 6%, and lifetime prevalence estimates from 7% to 14% (Essau, Conradt, & Petermann, 1999; Gren-Landell et al., 2009; Ranta, Kaltiala-Heino, Rantanen, & Marttunen, 2009; Shields, 2004; Väänänen et al., 2011; Wittchen, Stein, & Kessler, 1999). Prevalence estimates of depression in adolescence range from 3% to 10% (Kessler, Avenevoli, & Merikangas, 2001; Lewinsohn, Hops, Roberts, Seeley, & Andrews, 1993; Lewinsohn, Rohde, & Seeley, 1998; Roberts, Lewinsohn, & Seeley, 1995) and from 15% to 25% (Kessler & Walters, 1998; Lewinsohn et al., 1993), respectively. Comorbidity between social phobia and depression is common, and these two disorders have been a focus for quite many comorbidity studies (Beesdo et al., 2007; Bittner, Goodwin, & Wittchen, 2004; Lewinsohn, Zinbarg, Seeley, Lewinsohn, & Sack, 1997; Väänänen et al., 2011; Wittchen et al., 1999).

Based on several studies on the comorbidity of social phobia and depression, it has been hypothesized that social phobia may cause psychological and functional deterioration leading to depression (Kessler, Stang, Wittchen, Stein, & Walters, 1999; Merikangas et al., 1996) or there may be shared risk factors for both disorders leading to different phenotypes, which may vary over time (Angold, Costello, Farmer, Burns, & Erkanli, 1999). Among adolescents who suffer from social phobia, risk factors for subsequent depression have been found to be a number of feared situations, and pervasiveness, severity, and earlier onset of social phobia (Beesdo et al., 2007; Bittner et al., 2004; Chavira, Stein, Bailey, & Stein, 2004).

Low social support has been reported as a risk factor for adolescent psychiatric problems (Aseltine, Gore, & Colten, 1994; Ezzell, Swenson, & Brondino, 2000; Ritakallio, Luukkala, Marttunen, Pelkonen, & Kaltiala-Heino, 2010; Sheeber, Hops, Alpert, Davis, & Andrews, 1997). Blumenthal et al. (1987) reported in their original study using Perceived Social Support Scale (PSSS) that among adults probability to coronary artery disease was inversely related to the level of social support and that social support moderated the long-term health consequences for type As behavioural pattern (Blumenthal et al., 1987). Parental support has found to be a predictor of identity achievement in adolescent development (Sartor & Youniss, 2002). The interaction with social network may directly produce positive or negative psychological states being supportive or deprivating (Kawachi & Bergman, 2001).

Low social support has shown to be associated with adolescent depression in many studies (Bettge et al., 2008; Kaltiala-Heino, Rimpelä, Rantanen, & Laippala, 2001; Lewinsohn, Gotlib, & Seeley, 1997; Newman, Newman, Griffen, O'Connor, & Spas, 2007; Schraedley, Gotlib, & Hayward, 1999) and high social support has been found to have a buffering effect against the emergence of adolescent depression (Denny, Clark, Fleming, & Wall, 2004; Piko, Kovacs, & Fitzpatrick, 2009). Studies concerning the association between social support and social phobia are much less common. Theoretically, low perceived social support may lead an adolescent to insecurity in social situation and, thus, to social phobia. Perceiving high social support from family and low social support from friends may also lead an adolescent to turn more to home from social situations possibly leading to social phobia. Adolescents with social anxiety have shown to experience lower support from friends and classmates in a study about perceived support from friends, classmates, and family (La Greca & Lopez, 1998), and less support from friends in a study about perceived support from mother, father, and friends (McDonald, Bowker, Rubin, Laursen, & Duchene, 2010).

During adolescent development adolescents turn to seek support from peers instead of their family. In childhood, families are the strongest source of support followed by peers and teachers (Ezzell et al., 2000). Among children in prospective study from 1st grade to 10th grade, most common support provider was biological father followed by grandparents (Appleyard, England, & Sroufe, 2007). During adolescence, the perception of source of support has reported to turn from family to peers (Garnefski, 2000).

The sources of social support have been found to have different impacts on risk for social phobia and depression. Both low family and low peer support have been linked to depression in middle and late adolescence in most studies (Denny et al., 2004; Lewinsohn, Gotlib, et al., 1997; McDonald et al., 2010), while school-related support has played only a modest role in relation to depression (Piko et al., 2009). High social support from friends but not from family or teachers has been found to protect against social phobia (La Greca & Lopez, 1998; McDonald et al., 2010).

Previous research has reported gender differences in social phobia, depression, and social support. In epidemiological studies, the prevalence of social phobia has found to be higher among girls than boys (Gren-Landell et al., 2009; Shields, 2004; Wittchen et al., 1999). The prevalence of depression is higher among girls from adolescence onwards, and risk factors for depression differ by gender. Conflicts with family, negative cognitive style, and rumination have been found to be risk factors among females only (Hankin, 2009; Hankin & Abramson, 2001; Lewinsohn, Rohde, Seeley, Klein, & Gotlib, 2000). Females have a higher risk for recurrence of depression (Lewinsohn et al., 2000). Among females with earlier social phobia the onset of subsequent comorbid depression takes place in a shorter interval than among males (Beesdo et al., 2007). Females report higher levels of perceived social support than males (Bettge et al., 2008), especially received from outside the family (Katainen, Räikkönen, & Keltinkangas-Järvinen, 1999; La Greca & Lopez, 1998). Stress and social support have been more strongly associated with depression among girls than boys in some studies (Bettge et al., 2008; Kaltiala-Heino et al., 2001), but not in all (Denny et al., 2004). Low social support has been shown to have a more deleterious effect on social functioning among girls than among boys (La Greca & Lopez, 1998).

Social phobia and depression are highly comorbid with each other, but there is a lack of prospective population-based studies among adolescents about possible psychosocial shared risk factors for these disorders. To better understand the development of social phobia and depression and thus to improve preventive interventions and treatment for these under-recognized and under-treated disorders (Kessler et al., 2001; Lewinsohn et al., 1998; Shields, 2004; Wittchen et al., 1999), prospective population studies on the psychosocial risk factors for these disorders and their comorbidity are needed. Further, more research on gender-specific patterns of risk factors is needed to develop earlier and more precise identification of social phobia, depression, and comorbid social phobia and depression, and to develop specific interventions of these disorders for girls and boys. The aims of our study were (1) to explore if low perceived social support from different sources is a risk factor for social phobia, depression, and comorbid social phobia and depression in two-year follow-up separately among boys and girls; (2) to identify cross-sectional associations between depression and social phobia and perceived social support among girls and boys aged 15 and 17 years; and (3) to analyse gender differences in perceived social support, and its associations with social phobia and depression in middle adolescence.

Based on previous literature we expected that (1) low perceived social support – particularly from peers – is a risk factor for both depression and social phobia; (2) low perceived social support from family is an additional risk factor for depression; (3) low perceived social support is associated with current social phobia and depression, being most evident among those with comorbid disorders; and (4) the above mentioned effects are more prominent among girls than boys.

## Materials and methods

### Study samples and procedures

This study is part of an ongoing prospective cohort study, the Adolescent Mental Health Cohort (AMHC) Study, conducted in two Finnish cities, Tampere (200,000 inhabitants) and Vantaa (180,000 inhabitants). These cities were chosen because they represent well Finnish urban population. Tampere is the largest provincial centre outside the capital city area, and Vantaa is about the same sized site in the capital city area of Finland. Data collection, procedures, and measures of the study have been reported in detail elsewhere (Fröjd, Marttunen, Pelkonen, von der Pahlen, & Kaltiala-Heino, 2006, 2007). Briefly, at baseline survey 2002–2003 (T1), data were collected from all ninth graders (age 15) in all Finnish-speaking secondary schools in the two cities. Subjects completed a person-identifiable survey during a school lesson under the supervision of a teacher. Another opportunity to participate was offered in the school within a couple of weeks for students absent from school on the original survey day. For students not present on either occasion the questionnaires were sent by post twice, and if no reply was received, it was concluded that the student was not willing to participate. Thus, the participation rate for T1 data collection was excellent (94.4%), consisting 3278 adolescents (mean age = 15.5 years, SD = 0.39; 1609 girls, 1669 boys, 69% living in two-parent families). Follow-up data collection was conducted in 2004–2005 (T2). Eligible participants at T2 were students who had participated at T1. We organized school-based surveys as T1 in upper secondary schools and vocational schools. Surveys were sent by post to adolescents not reached through schools and to those who did not respond even by post, the same survey was offered by Internet (Fröjd et al., 2006).

A total of 2070 adolescents completed the survey at both T1 and T2. The response rate of the final sample was 63.1% (2070/3278). Of the respondents 56.6% were girls. The mean age at T2 was 17.6 years (SD 0.4). At T2 over 80% of the adolescents were full-time students (girls 89.6%, boys 82.0%, *p* < .001). Cases were excluded if more than three items of our measures were unanswered. If only three or less items were unanswered, missing values were replaced with the mean value of the subject's responses to the other items of the scale. Because of too many unanswered items, 32 subjects were excluded from the analyses, and the final sample consisted of 2038 subjects, 1154 girls and 884 boys. In the analyses, we always used all data available on the issues of interest, and therefore, the number of subjects varies slightly between separate analyses.

### Measures

*Depression (DEP)*. A Finnish modification of the 13-item Beck Depression Inventory (R-BDI) (Raitasalo, 2007) was used to assess depression (Beck & Beck, 1972; Beck, Rial, & Rickels, 1974). The R-BDI is a widely used self-report scale measuring the severity of depressive symptoms, and its reliability and validity are good (Bennet et al., 1997). The R-BDI has been shown to be appropriate for measuring depression in Finnish adolescents in population studies (Kaltiala-Heino, Rimpelä, Rantanen, & Laippala, 1999; Raitasalo, 2007). Each item is scored 0–3 according to the severity of the symptom. Sum scores of 13 items (range 0–39) were dichotomized to non/mild depression (scores 0–7), and moderate/severe depression (scores 8–39) (Beck & Beck, 1972). A cut-off score of 8 predicts a diagnosis of depression by structured interview Schedules for Clinical Assessment in Neuropsychiatry with good sensitivity (0.93) and specificity (0.88) (Fountoulakis et al., 2003).

*Social phobia (SP)*. To measure social phobia we used the Social Phobia Inventory (SPIN) (Connor et al., 2000). SPIN is a 17-item self-report questionnaire for measuring symptoms of social phobia. It is constructed on a five-point Likert-type scale which has previously shown good reliability and construct validity (Johnson, Inderbitzen-Nolan, & Anderson, 2006; Ranta, Kaltiala-Heino, Rantanen, Tuomisto, & Marttunen, 2007) for use among English-speaking and Finnish adolescents. For the Finnish translation of SPIN, a cut-off of 24 points has resulted in 81.8% sensitivity and 85.1% specificity in relation to a diagnosis of social phobia based on the Schedules for Affective Disorders and Schizophrenia for school-aged children – Present and Lifetime version (Kiddie-SADS-PL) diagnostic interview (Ranta et al., 2007).

*Social support*. The Perceived Social Support Scale-Revised (PSSS-R) was used to measure perceived social support from multiple sources. The PSSS-R was first presented by Blumenthal et al. (1987) in their study on the impact of social support to moderate the association between behaviour type and coronary artery disease. The PSSS-R measures people's subjective perceptions of social support and emotional closeness (Blumenthal et al., 1987). It contains 12 items on a 5-point Likert-type scale. Factor-analytically derived sum scores were used for addressing perceived support from family, friends, and significant others (each ranging 4–20). High sum scores indicate high perceived social support. PSSS-R sum scores were used as a continuous variable. The PSSS-R has been shown to be a useful method for assessing perceived social support among Finnish adolescents (Katainen et al., 1999). Reliabilities for the subscales were for girls and boys, respectively, *α* = 0.91 and *α* = 0.82 for family support; *α* = 0.93 and *α* = 0.91 for significant other support; and *α* = 0.89 and *α* = 0.84 for support from friends (Fröjd et al., 2006).

### Covariates

In the statistical analyses, the controlled covariates were age (calculated from the date of survey and date of birth), family structure (asked to select from items in questionnaire on question ‘do your family include?’: mother and father; mother and stepfather; father and step mother; only mother; only father; someone else caretaker; who. For present study we dichotomized the answers to living with both biological parents, if the first item was selected/living in some other family structure, if some of the other items was selected), both parents' highest educational qualification (asked to select from items in questionnaire on question: what is the highest educational level your father has completed/‘what is the highest educational level your mother has completed?’ Items to select were comprehensive school only, comprehensive school and vocational school, college and vocational school, and university examine. For the present study we dichotomized the answers to comprehensive school only, if the first item was selected/higher education, if some of the other items were selected), and externalizing symptoms, measured by Finnish version of the Youth Self Report (YSR) (Achenbach, 1991), at T1. We used externalizing scale of YSR as continuous sum scores. These covariates were chosen since previous studies have suggested that they have an impact on the main variables of interest in the present study (Costello, Swendsen, Rose, & Dierker, 2008; DeWit et al., 2005; Hankin, 2009; Hankin & Abramson, 2001; Kendler, Gardner, & Prescott, 2002, 2006; Wittchen & Fehm, 2001).

### Attrition

Compared to responders at both surveys, there were more boys (63.4% vs. girls 36.6%; *p* < .001) and subjects with depression at T1 (11.7% (dropouts) vs. 9.1% (responders), *p* = .020) among dropouts. There were no differences in response rate at T2 among those with or without social phobia at T1 (65.1% vs. 63.1%, *p* = .523). Dropout boys' perceived support from family at T1 was significantly lower than that perceived by boys also responding at T2 (Mann–Whitney test, *p* = .015). Social support from family among girls, and social support from friends and from significant other among both genders did not differ significantly between responders and dropouts. Father's or mother's highest educational status was more often comprehensive school only or lower among dropouts at T2 (father 18.9% vs. 15.1, *p* = .005%; mother 16.1% vs. 12.2%, *p* = .002).

### Statistical analysis

We formed four groups of our sample according to disorder status: adolescents without social phobia or depression (no SP/DEP) (SPIN score < 24, R-BDI score < 8), with social phobia and without depression (SP) (SPIN ≥ 24, R-BDI < 8), with depression and without social phobia (DEP) (SPIN < 24, R-BDI ≥ 8), and with both social phobia and depression (comorbid SP/DEP) (SPIN ≥ 24, R-BDI ≥ 8).

To explore current social support according to disorder status, because the distribution of scores of the PSSS-R was non-Gaussian, the Kruskal–Wallis test at a significance level of *p* = .05 was used. To analyse one-to-one differences between disorder groups, we used the Bonferroni-corrected Mann–Whitney test. Because we had 4 groups, we had 6 paired comparisons, meaning that statistical significance of *p*-level 0.05/6 = 0.008333 was used.

The statistical significance of gender differences was tested by the Mann–Whitney test at *p* = .05 level. We used medians instead of means as the parameter, because of the non-Gaussian distribution of PSSS-R scores.

In analyses of longitudinal associations between social support at T1 and disorder status at T2, we selected the sub-samples free of both disorders at T1 to control the possible confounding effect of baseline SP and DEP to the results. For analyses we used the Kruskal–Wallis test. To control for the effects of covariates – age, family structure, both parents' highest educational qualification, and external symptoms – we used binomial logistic regression analysis.

Statistical significance was tested two-tailed.

Data analyses were made using SPSS, version 16.0 (SPSS Inc., Chicago, Illinois, USA).

## Results

### Cross-sectional associations between current SP, DEP, or comorbid SP/DEP and perceived social support at age 15 and 17 years

In the Kruskal–Wallis test, low scores of all subscales of PSSS-R, family, friends, and significant others, as well as total scores on the PSSS-R were associated with having DEP, SP, or comorbid SP/DEP at both T1 and T2 among both genders (*p* < .001) (Tables 1 and 2).

**Table 1.** Current PSSS-R scores according to disorders at age 15 years among girls and boys.

Note: SP = Social phobia (SPIN ≥ 24, R-BDI < 8), DEP = depression (SPIN < 24, R-BDI ≥ 8), and comorbid SP/DEP (SPIN ≥ 24, R-BDI ≥ 8). *M* = median, SD = standard deviation. Statistical significance by the Bonferroni-corrected Mann–Whitney test: disorder groups vs. no SP/DEP group: **p < .001, *p < .00833; DEP or comorbid SP/DEP groups vs. SP group: ^++^ *p* < .001, ^+^ *p* < .00833; comorbid SP/DEP group vs. DEP group: ^¤¤^ *p* < .001, ^¤^ *p* <.00833. *p* of gender difference: (1), in no SP/DEP; (2), in SP; (3), in DEP; and (4), in comorbid SP/DEP groups.

**Table 2.** Current PSSS-R scores according to disorders at age 17 years among girls and boys.

Note: SP = Social phobia (SPIN ≥ 24, R-BDI < 8), DEP = depression (SPIN < 24, R-BDI ≥ 8), and comorbid SP/DEP (SPIN ≥ 24, R-BDI ≥ 8). *M* = median, SD = standard deviation. Statistical significance by the Bonferroni-corrected Mann–Whitney test: disorder groups vs. no SP/DEP group: ** = *p* < .001, * = *p* < .00833; DEP or comorbid SP/DEP groups vs. SP group: ^++^ *p* < .001, ^+^ *p* < .00833; Comorbid SP/DEP group vs. DEP group: ^¤¤^ *p* < .001, ^¤^ *p* < .00833. *p* of gender difference: (1), in no SP/DEP; (2), in SP; (3), in DEP; and (4), in comorbid SP/DEP groups.

In the Bonferroni-corrected Mann–Whitney test among girls at ages 15 and 17 years, perceived social support scores, both total score and all subscale scores, were highest in the no SP/DEP group, and lowest in the comorbid SP/DEP group. Perceived social support from family at both T1 and T2 surveys, and total score and significant other subscale of the PSSS-R at T2 were lower in the DEP group than in the SP group (Tables 1 and 2).

Similarly, in the Bonferroni-corrected Mann–Whitney test among boys at both ages, perceived social support was highest in the no SP/DEP group and lowest in the comorbid SP/DEP group. Among boys perceived support from family was lower in the DEP group than in the SP group at T1 and T2, but perceived social support from significant other at T2 was lower in the SP group than in the DEP group (Tables 1 and 2).

### Longitudinal associations between perceived social support at age 15 years and incidence of SP, DEP, or comorbid SP/DEP during follow-up

In analyses made by the Kruskal–Wallis test among adolescents in no SP/DEP group at T1, low perceived social support at T1 predicted DEP at T2 but not SP or comorbid SP/DEP at T2. Among girls a statistically significant association was found between low total score (*p* = .004), family subscale (*p* = .008), and friend subscale (*p* = .008) of PSSS-R and subsequent DEP. Among boys the same association was found between low total score (*p* = .005), family subscale (*p* = .008), and significant other subscale (*p* = .001) and subsequent DEP.

In logistic regression after controlling for covariates (age, family structure, both parents' highest educational qualification, and external symptoms), low total score of PSSS-R (odds ratio (OR) = 0.944, 95% confidence interval (C.I.) 0.906–0.984, *p* = .006), low perceived support from friends (OR = 0.897, 95% C.I. 0.837–0.990, *p* = .031), and from significant other (OR = 0.871, 95% C.I. 0.786–0.975, *p* = .009) at T1 predicted DEP at T2 among girls. Among boys low PSSS-R total score (OR = 0.959, 95% C.I. 0.921–0.999, *p* = .046) and low support from significant other (OR = 0.882, 95% C.I. 0.798–0.975, *p* = .014) predicted DEP at T2. Low perceived support from the family did not predict DEP among either gender. As in analyses made by the Kruskal–Wallis test, in logistic regression with covariates total score of PSSS-R, or scores of any of the subscales of the PSSS-R at T1 did not predict SP or comorbid SP/DEP at T2 among either gender (Tables 3 and 4).

**Table 3.** Perceived social support at age 15 years and risk for disorders at age 17 years among girls without social phobia or depression at T1.

			Disorder status at age 17 years		
			*n* = disorder/no disorder		
			SP	DEP	Comorbid SP/DEP
			*n* = 41/851	*n* = 30/851	*n* = 22/851
Girls' perceived social support at age 15 years	Total score	OR	0.986	0.944	0.964
		*p*	.103	.006	.143
		95% C.I.	0.954–1.018	0.906–0.984	0.918–1.012
	Family subscale	OR	0.974	0.918	0.953
		*p*	.601	.073	.441
		95% C.I.	0.880–1.076	0.837–1.008	0.844–1.077
	Friend subscale	OR	0.933	0.897	0.932
		*p*	.114	.031	.248
		95% C.I.	0.855–1.017	0.812–0.990	0.828–1.050
	Significant other subscale	OR	0.918	0.871	0.908
		*p*	.071	.009	.105
		95% C.I.	0.836–1.007	0.786–0.975	0.807–1.020

Note: OR of the association to different disorder groups compared to the no SP/DEP group at age 17 years, statistical significance, and C.I. at 95% level in logistic regression controlling covariates of age at T1, family structure at T1, mother's highest educational qualification at T1, father's highest educational qualification at T1, and externalizing symptoms at T1. SP = Social phobia (SPIN ≥ 24, R-BDI < 8), DEP = depression (SPIN < 24, R-BDI ≥ 8), and comorbid SP/DEP (SPIN ≥ 24, R-BDI ≥ 8).

**Table 4.** Perceived social support at age 15 years and risk for disorders at age 17 years among boys without social phobia or depression at T1.

			Disorder status at age 17 years		
			*n* = disorder/no disorder		
			SP	DEP	Comorbid SP/DEP
			*n* = 30/686	*n* = 14/686	*n* = 20/686
Boys’ perceived social support at age 15 years	Total score	OR	0.986	0.959	0.999
		*p*	.380	.046	.955
		95% C.I.	0.954–1.018	0.921–0.999	0.959–1.041
	Family subscale	OR	1.015	0.909	1.037
		*p*	.790	.121	.602
		95% C.I.	0.907–1.137	0.805–1.025	0.904–1.190
	Friend subscale	OR	0.945	0.933	0.985
		*p*	.176	.238	.772
		95% C.I.	0.870–1.026	0.832–1.047	0.888–1.092
	Significant other subscale	OR	0.960	0.882	0.987
		*p*	0.306	0.014	0.784
		95% C.I.	0.899–1.038	0.798–0.975	0.896–1.086

Note: OR of the association to different disorder groups compared to the no SP/DEP group at age 17 years, statistical significance, and C.I. at 95% level in logistic regression controlling covariates of age at T1, family structure at T1, mother's highest educational qualification at T1, father's highest educational qualification at T1, and externalizing symptoms at T1. SP = Social phobia (SPIN ≥ 24, R-BDI < 8), DEP = depression (SPIN < 24, R-BDI ≥ 8), and comorbid SP/DEP (SPIN ≥ 24, R-BDI ≥ 8).

### Gender differences in perceived social support and it's associations to SP, DEP, and comorbid SP and DEP

Analysed by the Mann–Whitney test in the total sample, girls reported significantly higher perceived support in all PSSS-R subscales except family subscale (*p* < .001 each) in both surveys. In the different disorder groups, girls reported higher perceived social support in all subscales, except family subscale at T1. At T2 in the DEP group there were no gender differences in PSSS-R total score or in any subscales of PSSS-R. Boys presented higher support from family in no SP/DEP and SP groups. There were no gender differences in family subscale in DEP or comorbid SP/DEP groups. Otherwise girls reported higher perceived support (Tables 1 and 2). Low perceived social support from friends at T1 predicted DEP at T2 only among girls (Tables 3 and 4).

## Discussion

Our main aim was to study low perceived social support as a risk factor for subsequent social phobia, depression, and comorbid social phobia and depression. Our hypothesis was supported only partly, because low perceived social support was predictive only for subsequent depression but not for social phobia or comorbid social phobia and depression.

Our findings of low perceived social support as a risk factor for depression are in accordance with those of earlier studies (Bettge et al., 2008; Heponiemi et al., 2006; Newman et al., 2007). After controlling for age, family structure, mother's and father's highest educational qualification, and externalizing symptoms at T1, low total perceived social support in total and support from significant other predicted depression at age 17 among both genders, and among girls low perceived support from peers predicted depression two years later. Different from earlier studies of middle adolescent samples, we found no association between low perceived social support from family and later depression (Aseltine et al., 1994; McDonald et al., 2010; Sheeber et al., 1997). Our different finding may be due to controlling for covariates, as controlling for them levelled out the predictive association between low family support and subsequent depression. Especially externalizing symptoms measured by YSR is important to control, because antisocial behaviour measured by YSR has found to be often comorbid with depression in earlier findings of analyses of our baseline sample (Ritakallio et al., 2010). Perceived social support had modifying effect to this comorbidity (Ritakallio et al., 2010).

Low perceived support from friends has been associated with later depression in some studies on mid-adolescent samples (McDonald et al., 2010; Schraedley et al., 1999) but not in all (Aseltine et al., 1994). In our study this was true among girls but not among boys. Thus support from friends seems to be more important for girls than for boys. Girls also have reported more perceived support from friends than boys in earlier studies as also in our study (Katainen et al., 1999; La Greca & Lopez, 1998). Girls may in general emphasize intimate dyadic relationships with friends, while boys may direct to larger peer groups, and friends have thus stronger meaning as source of support among girls than among boys (Facio & Batistuta, 2001). As low perceived social support from significant other was predictive to later depression among both genders, it seems that in middle adolescence both boys and girls need a close person to support them apart from their family and friends.

Low perceived social support from any source was not found to be a risk factor for social phobia without depression. Neither was low perceived family support nor low perceived support from significant other found to predict later social phobia in the study by LaGreca and Lopez (1998). In contrast to our findings, however, LaGreca and Lopez reported low perceived support from friends to be a predictor of subsequent social phobia (1998). The reason for this difference may be that our sample for risk factor analyses was free of both social phobia and depression at T1. As depression is associated with lower perceived social support in our study and in many earlier studies (Aseltine et al., 1994; Bettge et al., 2008; Hankin, 2009; Kaltiala-Heino et al., 2001; McDonald et al., 2010; Newman et al., 2007), and also associated with social phobia (Beesdo et al., 2007; Bittner et al., 2004; Lewinsohn, Gotlib, et al., 1997; Väänänen et al., 2011; Wittchen et al., 1999), it is of great importance to control for the effect of both of these disorders when studying risk factors.

Our finding that there was no association between low perceived social support and later comorbid social phobia and depression was unexpected, especially as there was an association between low perceived social support and later depression without social phobia. This finding may indicate, first, that comorbid social phobia and depression is more strongly linked to risk factors of social phobia than to risk factors of depression; second, that depression coexisting with social phobia develop differently compared to depression without social phobia; or third, that comorbid social phobia and depression is a qualitatively different disorder from social phobia or depression alone.

Low perceived social support has been associated with social phobia and depression in cross-sectional studies (Denny et al., 2004; Piko et al., 2009). As we hypothesized, the same was true in our study. All PSSS-R subscales were lower among affected than non-affected adolescents at ages 15 and 17 years. As could be expected, social support was lowest among those with both social phobia and depression and highest among those free from both disorders. Depressed adolescents perceived lower social support from their family than did adolescents with social phobia. This may be due to the nature of the disorders. Adolescents suffering from social phobia may turn more to their family to seek support than those suffering from depression. Contrary to what could be hypothesized due to the nature of these disorders, perceived support from outside the family was not lower among adolescents with social phobia than adolescents with depression. It may be that the depressed adolescent feels that nobody likes him/her and perceives less support from friends and significant other. An adolescent with social phobia may still have a good intimate relationship with a significant person and perceive support from that person.

As in earlier studies, girls reported higher perceived support than boys in all factors except family subscale (Bettge et al., 2008; Katainen et al., 1999; La Greca & Lopez, 1998). Family was an equal source of support for both genders in middle adolescence. This may indicate that girls reach out more outside the family for social support or that they utilize more support from outside the family than boys. Girls have found to be more relationship-oriented than boys (Gavin, Furman, & Furman, 1989). There were some gender differences in the patterns of how perceived support was associated with social phobia, depression, and comorbid social phobia and depression. This emphasizes the importance of studying girls and boys separately and taking gender into account when planning prevention and intervention programmes for adolescent suffering from social phobia and/or depression.

### Methodological considerations

The strengths of the present study are the longitudinal design and a large population sample. Compulsory comprehensive school covers more than 99% of adolescents until age 16 in Finland. The cohort may thus be considered broadly representative of the age group studied.

There was a good response rate in the AMHC baseline survey. However, dropout at T2 was relatively high. Attrition was more common among boys than girls and might affect gender differences in perceived social support found in our study. Non-responders had more often depression among both genders in the baseline survey and lower perceived social support from family among boys. Thus, this must be taken into account in the interpretation of the results of the current study. This may have affected our result indicating that low perceived support from family was not a risk factor for later depression among boys. It may also have affected our finding of no gender differences in perceived social support from family. However, these assumptions are contra versed as there were no gender differences in cross-sectional analyses in the association between depression and perceived social support from family already at baseline. In addition, subjects without either disorder at baseline were used in longitudinal analyses. van Loon, Tijhuis, Picavet, Surtees, and Ormel (2003) have stated that even high levels of attrition may not necessarily affect the associations studied in health surveys (2003).

One limitation of this study is the lack of diagnostic interviews. However, the measures of the present study have previously been used in large community samples of adolescents. Adolescents have also been shown to be able to reliably report their symptoms in psychological disorders (Smith, Pelham, Gnagy, Molina, & Evans, 2000).

Another limitation is the lack of control for other possible disorders, which may affect our main results. It must be kept in mind that only a limited range of covariates can be controlled for in large epidemiologic studies. However, we controlled for externalizing symptoms, which is seldom done in studies on depression and anxiety.

As scores of PSSS-R were distributed in a non-Gaussian way, the use of logistic regression may not give accurate results. However, if the effects of covariants are to be controlled, logistic regression must be used. In large samples it could be reliably used (Tabachnick & Fidell, 2005).

## Conclusions

Low perceived social support is not a shared risk factor for social phobia and depression in middle adolescence. Low social support predicts depression, but is not an antecedent of social phobia. Adolescents suffering from social phobia and depression perceive low support from several sources. Perceived social support should be a part of the clinical evaluation of adolescents suffering from these disorders and of planning preventive and intervention strategies. This may be done by promoting possibilities for supportive relationships and enhancing one's capability to utilize one's relationships as sources of social support. More research on risk factors and on the development of comorbid social phobia and depression is needed, as comorbid disorder was differently associated with social support than depression alone.
